# Experimental research and application of drilling and blasting with directional damage-reduction shaped charge

**DOI:** 10.1038/s41598-024-60072-z

**Published:** 2024-04-25

**Authors:** Kaixing Liu, Yi ping Zhang, Yifei Peng, Lianhua Wu

**Affiliations:** 1 Guizhou University School of Mining, Guiyang, 550000 Guizhou China; 2 Guizhou Yihe Technology Co., Ltd, Guiyang, 550000 Guizhou China; 3 Emergency Management Bureau of Guiyang Economic and Technological Development Zone, Guiyang, 550000 Guizhou China

**Keywords:** Cumulative blasting, Slit blasting, Model test, Blast crack analysis, Strain analysis, Environmental sciences, Space physics, Energy science and technology, Engineering

## Abstract

In this research, a directional reduction charging structure was proposed to solve the problems caused by drilling and blasting method such as serious damage to surrounding rocks, working face low contour flatness and serious over-under break of root base c. Drilling and blasting tests, numerical calculations and field applications were designed and performed for the verification of the blasting advantages of charge structure. Test results showed that the peak positive strain along the protection direction of directional protection shaped charge was significantly smaller than that of ordinary charge, where PVC material presented the strongest effect such that the peak positive strain of specimen 1 at measuring point 4 (protection direction) was only 0.27 times that at measuring point 9 (non-protected direction). Numerical simulations indicated shaped jet formation, damage-reduction and charge penetration process and obtained the force law of cement target plate. Experimental results revealed that application of charge in tunnel controlled blasting achieved a clear controlling effect on contour line excavation. Compared with ordinary smooth blasting method, all technical indicators of the developed method were improved such that half hole mark rate was increased by about 33% and the amount of over-under break was decreased by about two times. Research results are of certain significance for the stability of surrounding reserved rocks and formation of roadway in blasting engineering and the developed method was found to be applicable to mining, shaft excavation and other projects.

## Introduction

Blasting operations are extensively being applied in infrastructure projects due to their economical and efficiency advantages; however, they might cause serious damages to surrounding rocks ^1^. Only a fraction of explosive blasting energy is applied to break rocks and most of it acts on reserved rock mass in the form of high-temperature air and shock waves, damaging reserved rock mass. Research on rock blasting mechanism has resulted in the development of blasting stress wave damage, explosion products damage, and blasting stress wave and explosion products combined damage theories ^2–4^. The ideal rock-breaking effect consists of utilization of high explosive energy and causing little damage to reserved surrounding rocks. Therefore, accurate control of blasting surrounding rocks and reduction and prevention of blasting energy propagation to reserved rock mass have always been the focus of researchers and engineers.

Rock directional fracture controlled blasting technology has been constantly improved by traditional smooth blasting method. Through continuous theoretical analyses and experimental explorations, the principle of directional fracture controlled blasting has been greatly developed ^5–8^. Rock directional fracture controlled blasting technologies could be classified into three classes: slotting blasting, energy-gathered blasting and slitting blasting. Slotting blasting applies special drilling tools for the formation of symmetrical *V*-shaped notches in blast holes, thereby changing blast hole shape and directionally concentrating explosive energy to break the rock, causing stress concentration in specific directions around blast holes. The *air wedge* formed by slotting generates an initial directional crack in a specific direction at blasting moment ^9^. As early as 1905, Alisakov ^10^ suggested axially slotting on borehole walls to guide fractured rocks. However, due to the limitations of construction technology at the time, this approach did not attract much attention. Foster et al. ^11^ first developed a blasting method for prefabricating *V*-shaped notches in borehole walls to control crack propagation direction in the rock after blasting. In 1960s, Langefors et al. ^12^ performed comparative blasting tests and found that circular boreholes were not as effective as *V*-shaped boreholes in controlling the generation and expansion direction of cracks. Yang et al. ^13^ applied a hyper dynamic strain testing system for comparing the stress distributions of ordinary and slotting blasting. Organic glass target experiments indicated a big stress distribution difference between the two and slotting was found to strengthen stress along this direction. Instantaneous pressure relief resulted in the appearance of a low stress zone appeared along non-slotting direction. Furthermore, caustics experiment system was also applied for quantitative evaluation of the blasting effect of slotting blasting. Research showed that the cracking toughness of rock in slotting blasting was 0.54 times that in traditional blasting ^14^. Shaped blasting was consisted of attaching a metal shape charge to a specific position of the charge and the shaped metal jet generated by the blasting acted on rock hole to form initial orientation cracks. Large-scale experiments and comprehensive application of shaped blasting began in military industry during World War II. Held et al. ^15,16^ performed armor penetration research using shaped jets. ClarkJ et al. ^17^ carried out blasting tests with shaped charges and applied flash X-ray photography to record shaped charge blasting fluctuation process. By the end of 1940s, Birkhoff et al. ^18^ and other researchers made great breakthroughs in theoretical research on explosive action mechanism of shaped charges and independently developed a mechanical analysis model for shaped charges. Bjarnholt et al. ^19^ used linear shaped charges in engineering rock blasting, which opened up a new field for the research and application of engineering blasting. Luo et al. ^20^ analyzed and designed relevant parameters for shaped charges and performed model tests to show that blasting energy was effectively concentrated along energy gathering direction. Rock directional blasting technology with shaped charges was found to be a better tunneling method. Ma et al. ^21^ applied ANSYS/LS-DYNA software for the simulation of crack propagation law of rock under shaped charge blasting. Meng et al. ^22^ used the same software to investigate shock wave and stress distribution rules of underwater blasting using shaped charge technology. Hussain et al. ^23^ used ANSYS/LS-DYNA software for comparative analysis of the energy-gathered effects of double-layer energy-gathered tubes and ordinary shaped charges. Research results showed that when charge length was smaller, ordinary energy-gathered tubes were more advantageous. McDonald ^24^ employed ALE algorithm in LS-DYNA software for the simulation of linear shaped charge blasting process and proved that shaped jet after blasting could form directional cracks. Kang ^25^ and Zhou et al. ^26^ evaluated the influences of the shape, material and angle of shaped charges on energy-gathered blasting penetration effect. Slitting blasting guided the propagation of blasting products using charge structure slitting shell, so that the blasting energy acted preferentially on rock holes along slitting direction, generating initial directional cracks on borehole walls along slitting direction. Also, the goal of directional rock fracture was finally achieved with the expansion of cracks. Seam blasting has been extensively applied in several engineering fields ^27–29^. Fourney et al. ^30–32^ wrapped charges with slitting tubes for the first time in blasting experiments, performed blasting experiments using slit charges, and conducted comparative experiments on metal slitting shells and ordinary blasting methods. Jiang ^33^ investigated the effects of pipe material and slitting size on crack propagation direction. Furthermore, high-speed Schlieren experiments were performed to investigate shock wave and explosion products propagation properties in slitting blasting ^34^ and slitting charge parameters in smooth blasting were optimized ^35^. By performing several indoor and mine field experiments, Gao et al. ^36^ investigated the influences of directional fracture blasting of charges with different shapes. Then, they discovered slitting charge hyperiority for rock directional fracture blasting and presented corresponding recommended parameters and blasting technology for slit charges. Yang et al. ^37^ simulated slit charge single-hole blasting process and systematically analyzed the complete action process of slitting shell under blasting load. Ma et al. ^38^ applied LS-DYNA for the simulation of rock cracks blasted by slit charges according to JH C constitutive model. Wang et al. ^39^ performed numerical simulations to evaluate the blasting effects of slit charge blasting on organic glass plates and compared and verified the results obtained from dynamic caustics tests and numerical simulations. Previous research works have investigated slotting blasting, energy-gathered blasting and slitting blasting and have achieved excellent results. The above technologies have addressed the problem of uncertain cracking directions in rock blasting, but damage to reserved rock masses due to explosive products has not been completely solved yet.

To sum up, in this research, a directional damage-reduction shaped charge was developed which could optimize explosive energy distribution and guide explosive energy release along the direction of the rock to be blasted, thereby protecting reserved rock masses. Since PVC pipes, EVA foam, and high-elastic shock-absorbing PU boards possess shock-proof and buffering characteristics, these materials were selected as damage reduction materials for this charge structure. This research combined the results of drilling and blasting model tests, numerical simulations, theoretical derivation and field application methods using high-speed cameras and ultra-dynamic stress and strain gauges to explore the blasting damage mechanism of charge and investigate the blasting effects of charge with different damage reduction materials, which was of practical value for engineering blasting.

## Drilling and blasting experiment

### Experimental equipment and plan

Drilling and blasting model experiments were carried out to explore the contributions of directional damage-reduction shaped charges in directional fracturing and surrounding rock protection during drilling and blasting. Comparative tests were performed by using charges made of PVC, EVA foam and PU board damage-reduction materials. A high-speed camera was applied to record protection and non-protected directions of specimens, capture the data on explosion products diffusion during blasting process, and collect blasting strain data by arranging strain gauges on concrete surfaces and sides. Finally, comprehensive analyses were performed on high-speed camera and strain data as well as the intuitive effects of concrete model to explain charge blasting mechanism.

#### Structural design of charge

**Figure 1:**
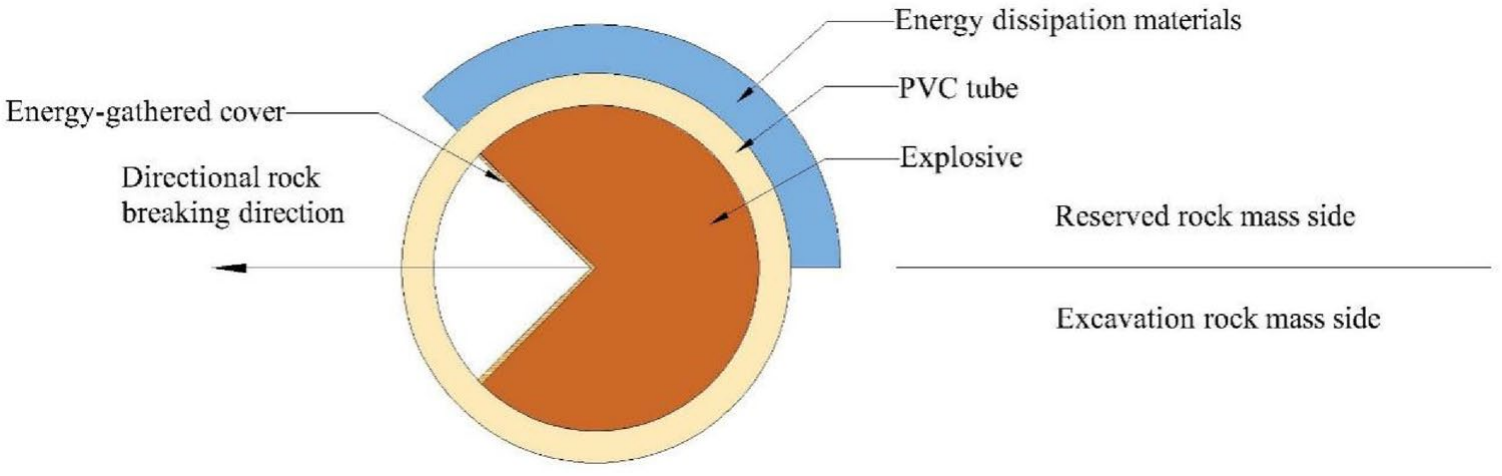
Structural diagram of charge.

**Figure 2:**
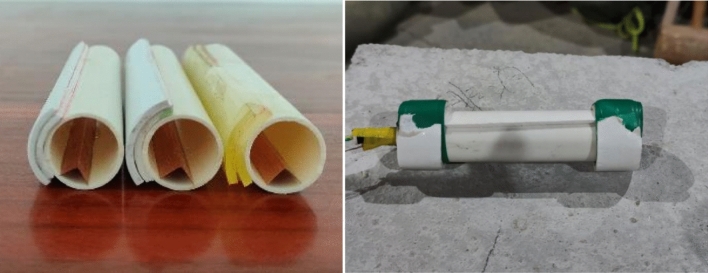
Production of charge.

Shaped charges and a wall-protecting material were applied in experiments. Shaped charge shell was made of PVC with 20 mm outer diameter, 18 mm inner diameter, and 100 mm length. The energy-gathered cover was 0.1 mm thick, 100 mm long, and 10 mm wide and energy-gathered cover angle was 90°. Emulsion explosives were applied in tests. There was a critical diameter for emulsion explosive detonation; therefore, it was designed as a single-energy-gathered structure. Charge structure is illustrated in Fig. 1, the physical charge is shown in Fig. 2 (Table 1).

**Table 1:** Test results of different dissipation materials 4 mm in thickness.

Number	Borehole diameter (mm)	Tube outer diameter (mm)	Tube inner diameter (mm)	Dissipation material thickness (mm)	Dissipation material
1	32	20	18	4	PVC pipe
2	32	20	18	4	EVA foam
3	32	20	18	4	PU board

#### Specimen design

**Figure 3:**
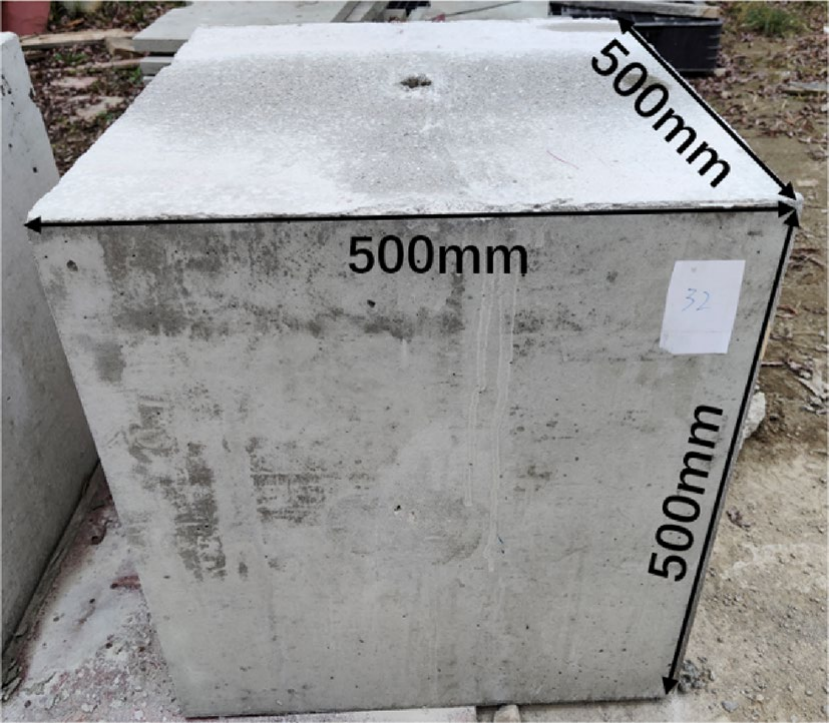
Diagram of concrete test block.

In this research, specimens with dimensions 500 mm × 500 mm × 500 mm were poured for drilling and blasting tests. Specimen pouring process was performed in three steps: mold making, borehole reserving, pouring and maintenance. The specimen after maintenance is illustrated in Fig. 3.

#### Experimental equipment and measuring point layout

##### Hyper dynamic strain testing system

The hyper dynamic strain testing system (H59392, Jiangsu Donghua Testing Co., Ltd., China) applied in this research was mainly consisted of hyper dynamic strain gauges, amplifiers, and strain gauges. The highest sampling frequency of DH5939 was 10 MHz. It was mainly employed to collect, store, display, and process voltage signals and was used with DH3842 amplifier.

##### High-speed photography

PhantomV710 high-speed photography had maximum shooting speed of 1.4 million FPS and full frame of 1280 × 800@7530 FPS. High-speed photography mainly monitored instantaneous energy release of blasting through by observing the hole outside the blasting tower to provide a basis for the analysis of test results.

##### Measuring point layout

**Figure 4:**
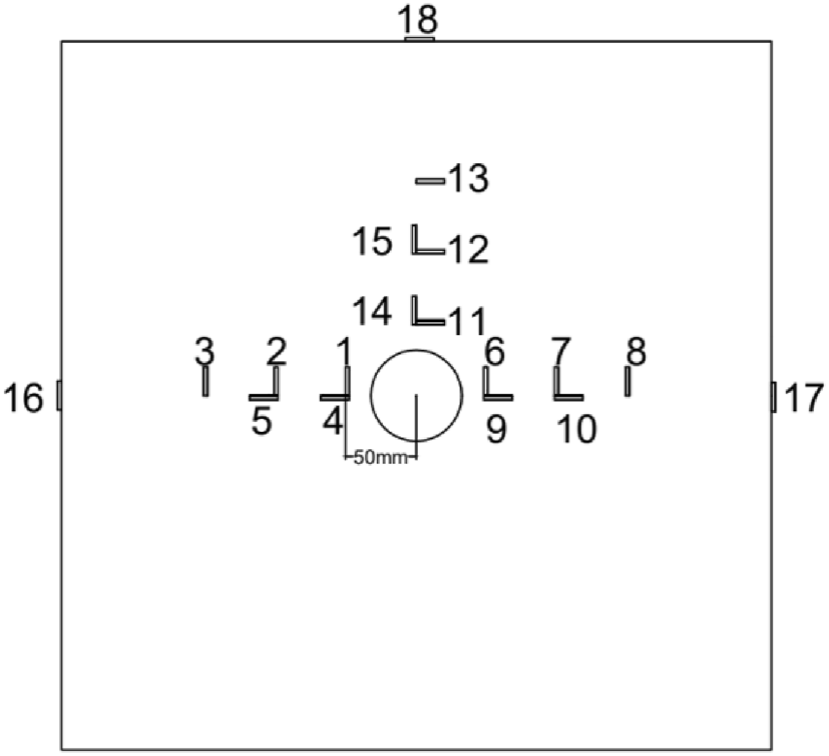
layout of strain gauge point.

Hyper dynamic strain testing system: stress strain gauges were installed 50 mm away from the boreholes along energy gathering, protected and non-protected directions on the upper surface of each concrete block with a spacing of 50 mm. A stress strain gauge was attached on the center of each concrete side wall along energy-gathered, protection, non-protected directions. Strain gauge point layout is illustrated in Fig. 4.

**Figure 5:**
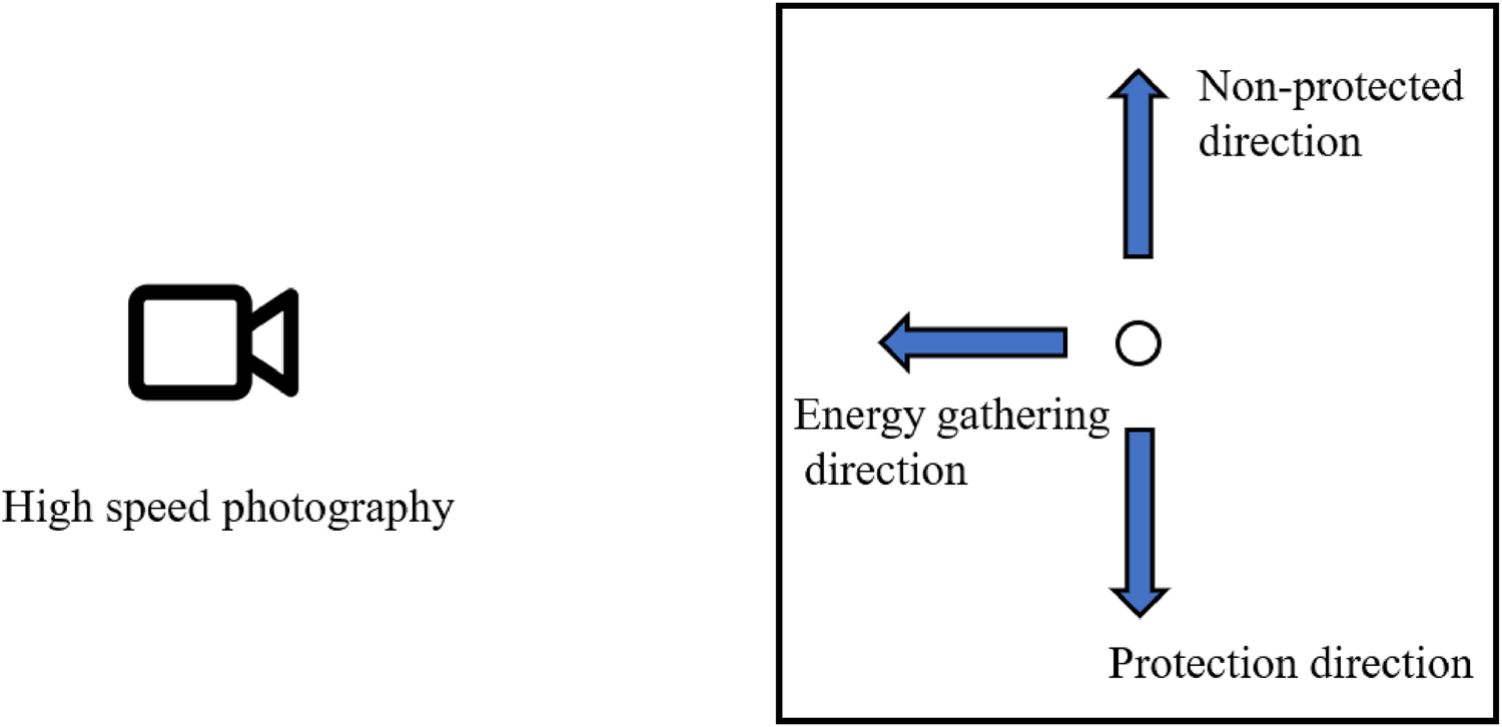
Observing image of high-speed photography.

High speed photography: the entire blasting processes of specimens were recorded. High-speed photography was performed along the direction of energy gathering. Protected and non-protected directions of specimens were and observed and recorded from energy-gathered direction. Damage-reduction effect was evaluated by observing explosion products diffusion speed. Frame rate during recording was 30,000 FPS and shooting position is illustrated in Fig. 5.

### Analysis of test results

#### Analysis of explosion products diffusion properties

**Figure 6:**
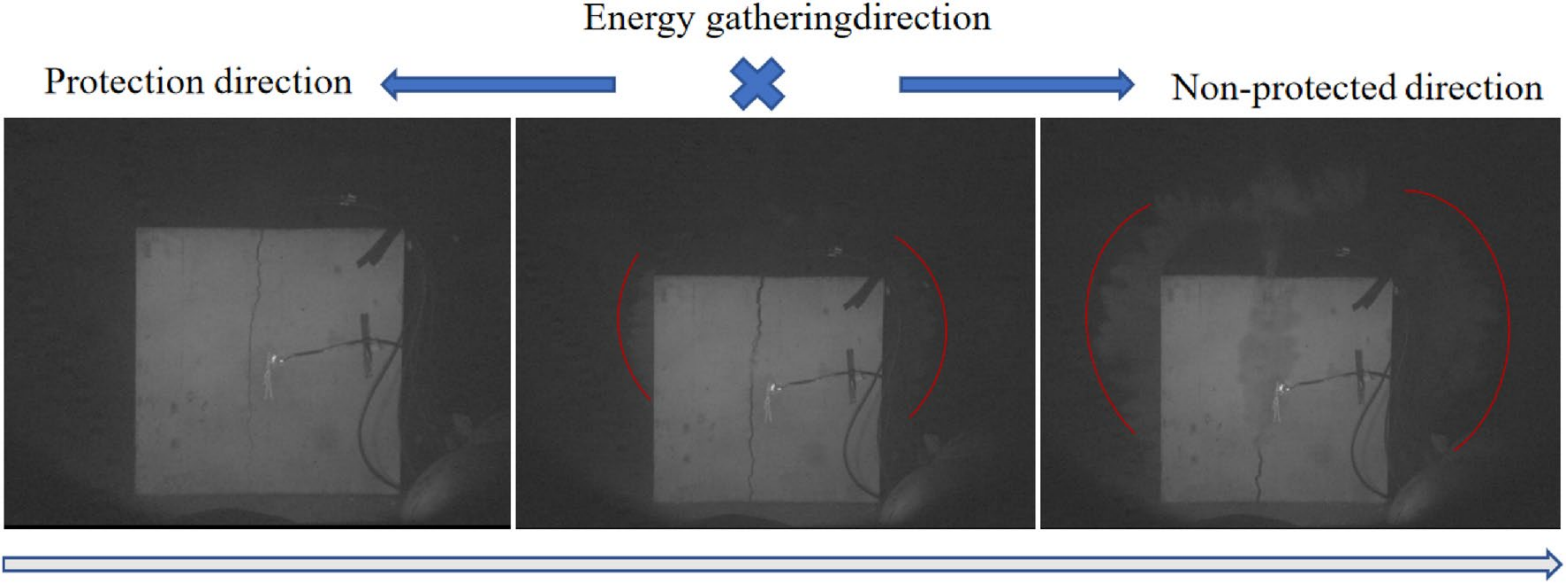
Blasting process of specimen 1.

**Figure 7:**
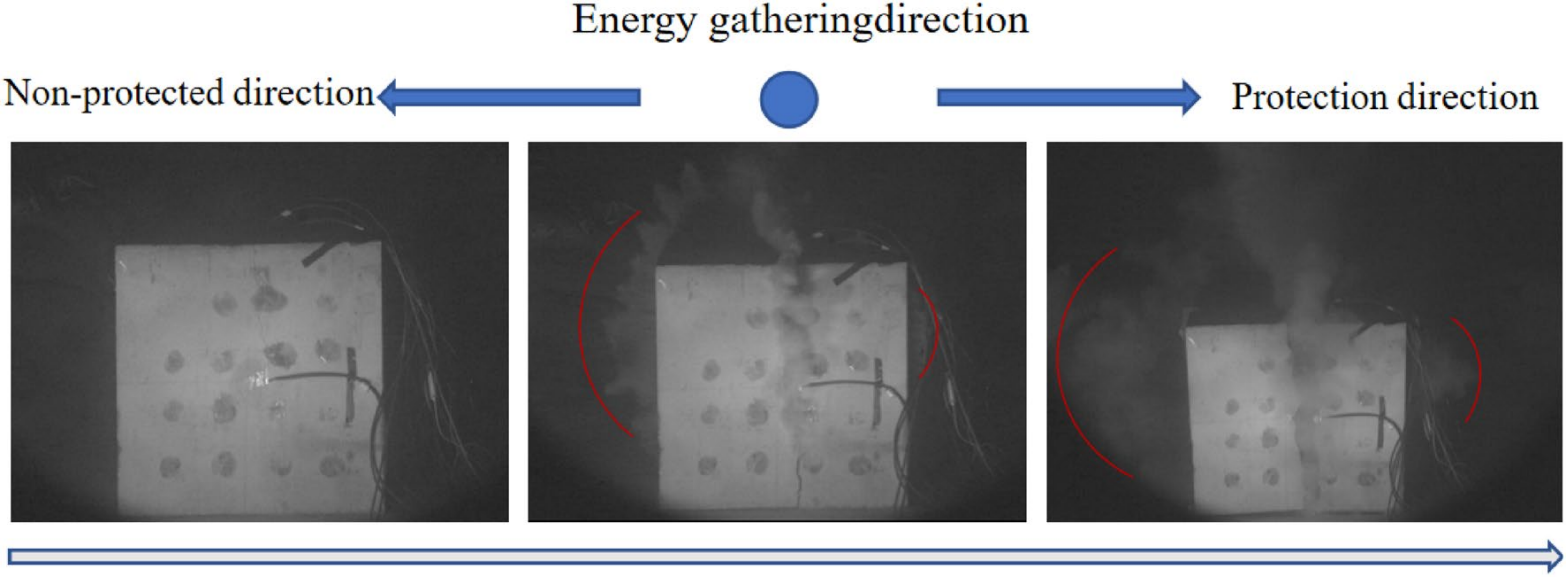
Blasting process of specimen 2.

**Figure 8:**
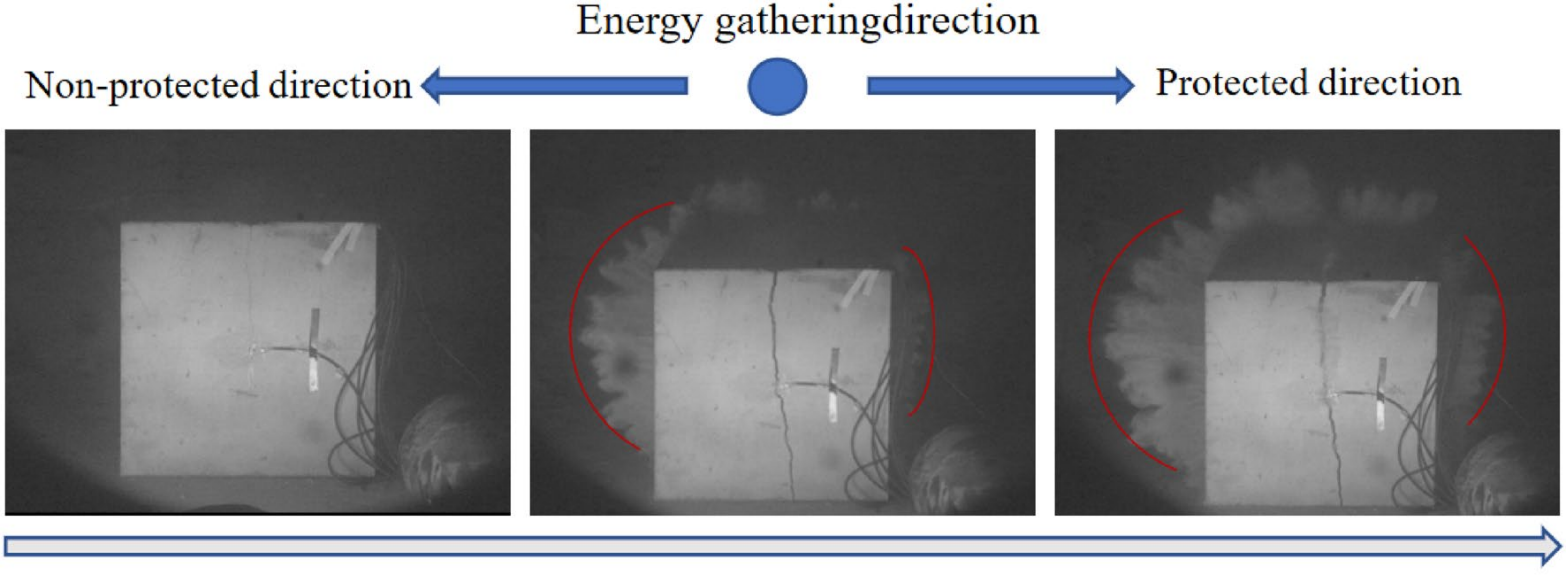
Blasting process of specimen 3.

Specimens 1, 2 and 3 were subjected to directional damage-reduction shaped charge blasting tests with dissipation materials of PVC pipe, EVA foam, and PU board, respectively, and their blasting processes were recorded by high-speed photography, as illustrated in Figs. 6, 7, and 8. In Fig. 6, the left side of the specimen was considered as damage-reduction (protection) direction and its right side was non-damage-reduction (non-protected) direction. The direction of energy gathering is perpendicular to the paper inward. In Figs. 7 and 8, however, the left side showed non-protected direction and the right side presented protection direction. The direction of energy gathering is perpendicular to the paper surface outward. It was seen from Figs. 6, 7, and 8 that cracks and explosion products appeared along the energy-gathered directions of three specimens. In each specimen, explosion products diffusion speed along non-protected direction was higher than that along protection direction and explosion products diffusion range along non-protected direction was larger than explosion products expansion range along protection direction. It was speculated that the reason for this phenomenon was that charge produced blasting products at blasting moment and explosive products along non-protected direction directly acted on rock wall. Along protection direction, explosive products first acted on the dissipation material and then on the rock wall. Protective material weakened the effects of explosive products.

#### Analysis of model crack characteristics

**Figure 9:**
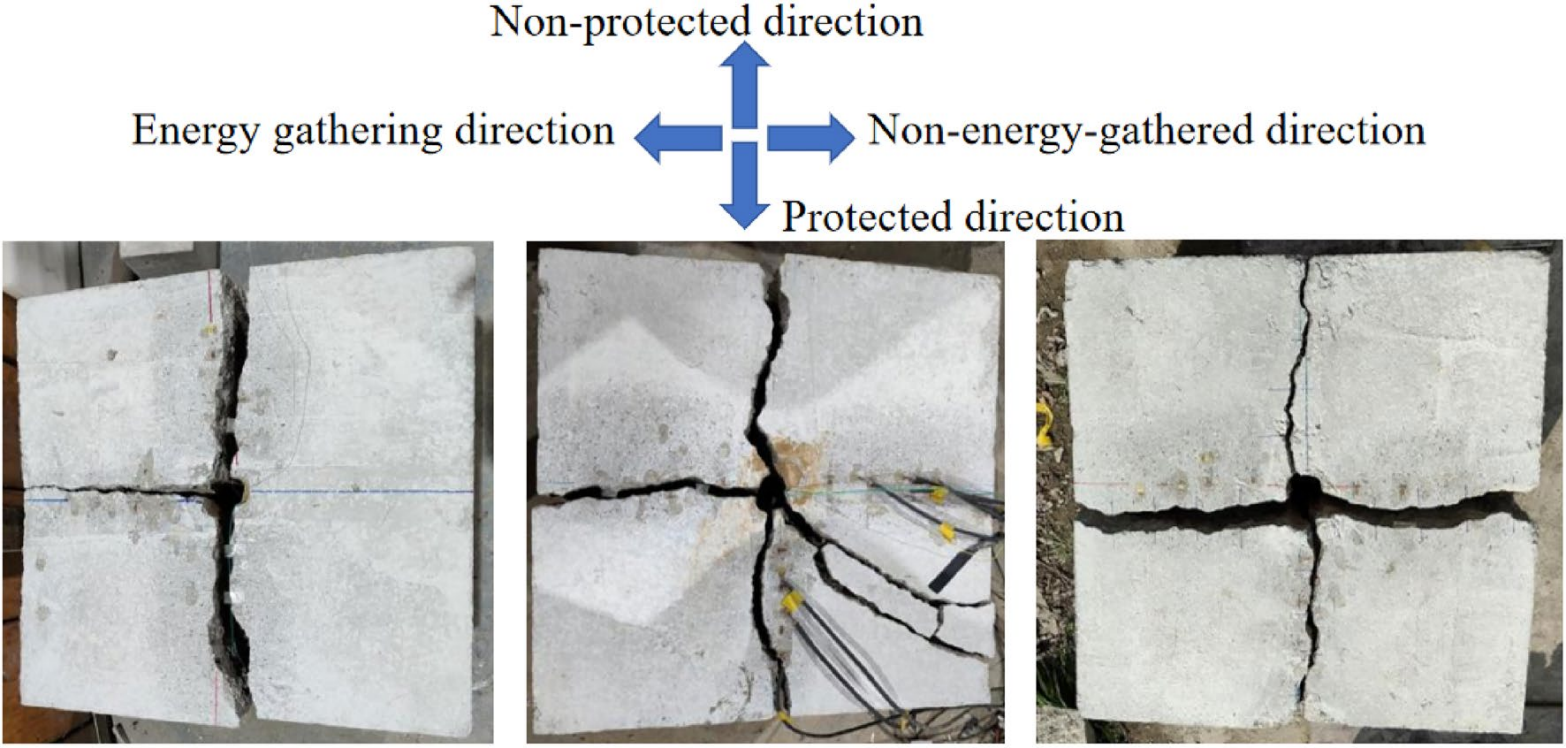
Model crack characteristic diagrams of (**a**) crack specimen 1, (**b**) specimen 2, and (**c**) specimen 3.

Model crack characteristics are illustrated in Fig. 9. In terms of post-blasting effect, all directional damage-reduction shaped charges formed cracks along energy-gathered cover direction, indicating that the structure of directional damage-reduction shaped charge presented directional controlled fracture effects. No cracks were observed in specimens 1 and 2 along non-energy-gathered direction, but in specimen 3 with PU board as dissipation material of charge, cracks appeared along non-energy-gathered direction. Hence, it could be concluded that the dissipation ability of PU board was weaker than PVC and EVA foam. Specimens 1–3 presented cracks along both protection and non-protected directions. In specimen 2, cracks along protection direction were offset and penetrated in certain arcs. This could possibly be because dissipation material was not close to tube or when specimen was poured, there were bubbles inside specimens which affected crack penetration. Combined with the data recorded by high-speed camera, explosion products distribution at the same time was analyzed to explore whether dissipation material hindered explosion products outward diffusion, indicating that charge structure had certain dissipation ability and presented protective effect on borehole wall.

#### Analysis of strain change characteristics

**Figure 10:**
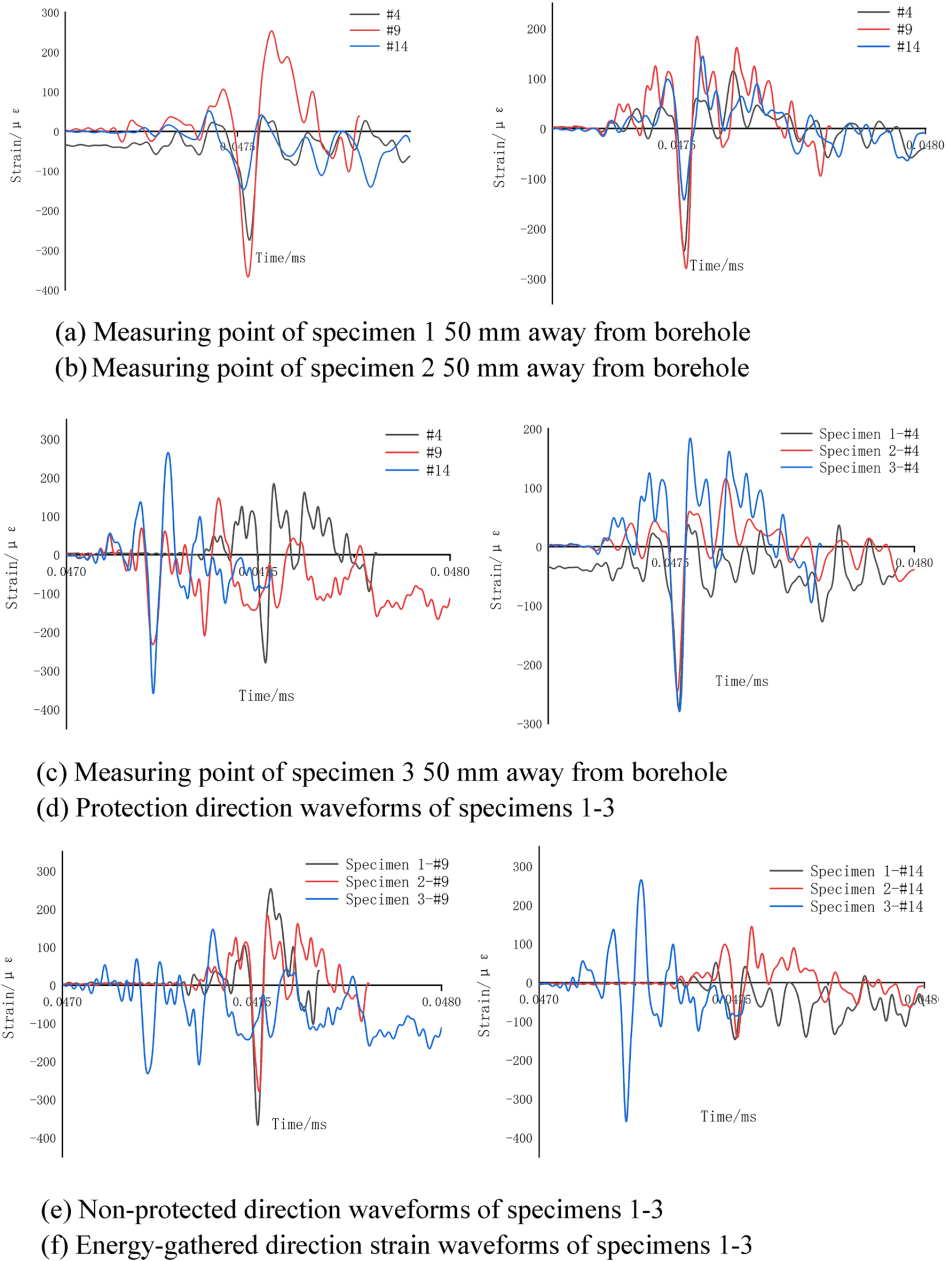
Strain waveform diagrams.

Hyper dynamic strain testing system was applied for collecting strain data during blasting process and the data obtained at the measuring point 50 mm away from borehole center was processed. Figure 10 shows the processed waveform.

From Fig. 10a–c, it was seen that at a distance of 50 mm away from borehole center, strain waveform overall variation laws of the above three specimens were consistent. Peak strain times along the three directions presented in Fig. 10a,b were basically the same. In Fig. 10c, strain peak appeared first along non-protected and energy-gathered directions and strain peak appeared later along protected direction. The strain peaks of specimens 1 and 2 followed the order of non-protected direction > energy-gathered direction > protection direction.

It was seen from Fig. 10d–f that along protection direction: overall waveform variation laws of the above three specimens were basically the same and the strain peak of specimen 3 was greater than those of specimens 2 and 1. Along energy-gathered direction, the strain peak of specimen 3 appeared earlier and larger than those of specimens 2 and 1. Along non-protected direction, the waveform pattern of specimen 3 had poor regularity and that of specimen 2 was generally consistent with that of specimen 1 with little difference in peak values.

Strain peaks of specimens 1–3 were obtained on a DH5960 hyper dynamic strain testing and analysis system at a distance of 50 mm from borehole center. Tables 2, 3, 4, 5, 6, 7, 8, 9, and 10 summarize the strain peaks of each measuring point.

**Table 2:** Strain peak at different measuring points of specimen 1.

Strain peak (με)	Distance from borehole center (mm)	50
	Measuring point	4/9/14
Protection direction	Positive	69.38
	Negative	242.35
Non-protected direction	Positive	253.1
	Negative	367.35
Energy-gathered direction	Positive	51.58
	Negative	147.49

**Table 3:** Strain peak values at different measuring points of specimen 2.

Strain peak (με)	Distance from borehole center (mm)	50
	Measuring point	4/9/14
Protection direction	Positive	115.04
	Negative	245.15
Non-protected direction	Positive	184.08
	Negative	279.54
Energy-gathered direction	Positive	144.34
	Negative	142.61

**Table 4:** Strain peak values at different measuring points of specimen 3.

Strain peak (με)	Distance from borehole center (mm)	50
	Measuring point	4/9/14
Protection direction	Positive	65.28
	Negative	255.44
Non-protected direction	Positive	146.72
	Negative	232.45
Energy-gathered direction	Positive	264.15
	Negative	358.95

**Table 5:** The relevant material parameters of explosive.

${\rho }_{e}$(kg/m^−3^)	VOD (m/s)	${E}_{e0}$(GPa)	${P}_{CJ}$	${A}_{e}$	${B}_{e}$	${R}_{1}$	${R}_{2}$	$\omega$
1320	6690	7.38	16	5.86 × 10^2^	21.6	5.81	1.77	0.282

**Table 6:** The relevant material parameters of copper charge liner.

$\rho$(g/cm^−3^)	C	${S}_{1}$	${S}_{2}$	${S}_{3}$	${\gamma }_{0}$	$a$	${E}_{0}$	${V}_{0}$
8.96	0.46	1.489	0	0	2.02	0.47	0	1

**Table 7:** The relevant material parameters of PVC.

$\rho$(kg/m^−3^)	E (Gpa)	$\mu$	${\sigma }_{y}$(MPa)	${E}_{tan}$(GPa)	BETA	C	P	FS
1300	3	0.25	22	0	0	252	5.96	0

**Table 8:** The relevant material parameters of air.

$\rho$(kg/m^−3^)	E_a0_ (J/m^3^)	${\gamma }_{\alpha }$	${C}_{0}$	${C}_{1}$	${C}_{2}$	${C}_{3}$	${C}_{4}$	${C}_{5}$	${C}_{6}$
1.29	2.5 × 10^5^	1.4	0	0	0	0	0	0	0

**Table 9:** The relevant material parameters of concrete target plate.

$\rho$(kg/m^−3^)	Shear modulus (GPa)	$A$	$B$	$C$
2180	18.75	0.79	1.60	0.007

**Table 10:** Blasting parameters of field test.

Parameter	Ordinary blasting	Blasting with directional damage-reduction shaped charge
Borehole diameter (mm)	40	40
Charge diameter (mm)	32	28.2
Radial uncoupling coefficient	1.25	1.42
Single-hole charge (KG)	0.6	0.45
Blasting mode	Reverse blasting	Reverse blasting
Charge mode	continuous charge	continuous charge

The strain data obtained for all directions for specimen 1 at a distance of 50 mm from borehole center were investigated and the following results were obtained for different directions: protection direction: the peak values of positive and negative strain at measuring point 4 were 69.38 με and 242.35 με, respectively. Non-protected direction: the peak values of positive and negative strain at measuring point 9 were approximately 253.1 με and 367.35 με, respectively. Therefore, the peak values of positive and negative strain at measuring point 4 were only 0.27 and 0.66 times those at measuring point 9, respectively.

The strain data obtained for all directions for specimen 2 at a distance of 50 mm from borehole center were analyzed and the following results were obtained for different directions Protection direction: the peak values of positive and negative strain at measuring point 4 were 115.04 με and 245.15 με, respectively. Non-protected direction: the peak values of positive and negative strain at measuring point 9 were approximately 184.08 με and 279.54 με, respectively. Therefore, peak values of positive and negative strain at measuring point 4 were only 0.62 and 0.88 times those at measuring point 9, respectively.

The strain data obtained for all directions for specimen 2 at a distance of 50 mm from borehole center was explored and the following results were obtained for different directions. Protection direction: the peak values of positive and negative strain at measuring point 4 were 65.28 με and 255.44 με, respectively. Non-protected direction: the peak values of positive and negative strain at measuring point 9 were approximately 146.72 με and 232.45 με, respectively. Hence, the peak values of positive and negative strain at measuring point 4 were only 0.44 and 1.09 times those at measuring point 9, respectively.

According to the above comprehensive analysis results, dissipation material effectively absorbed blasting energy and prevented explosive products from directly impacting reserved rock masses. Hence, the strain peak along protection direction appeared later and was smaller than that along non-protected direction. The peak values of positive and negative strain at measuring point 4 in specimen 1 were only 0.27 and 0.66 times those at measuring point 9, respectively. The protection effect of specimen 1 was higher than those of specimens 2 and 3. It could be concluded that among the three dissipation materials of PVC, EVA foam and PU board, PVC had the strongest protection effect, followed by EVA foam and PU board.

## Numerical simulation of penetration process

### Calculation model

**Figure 11:**
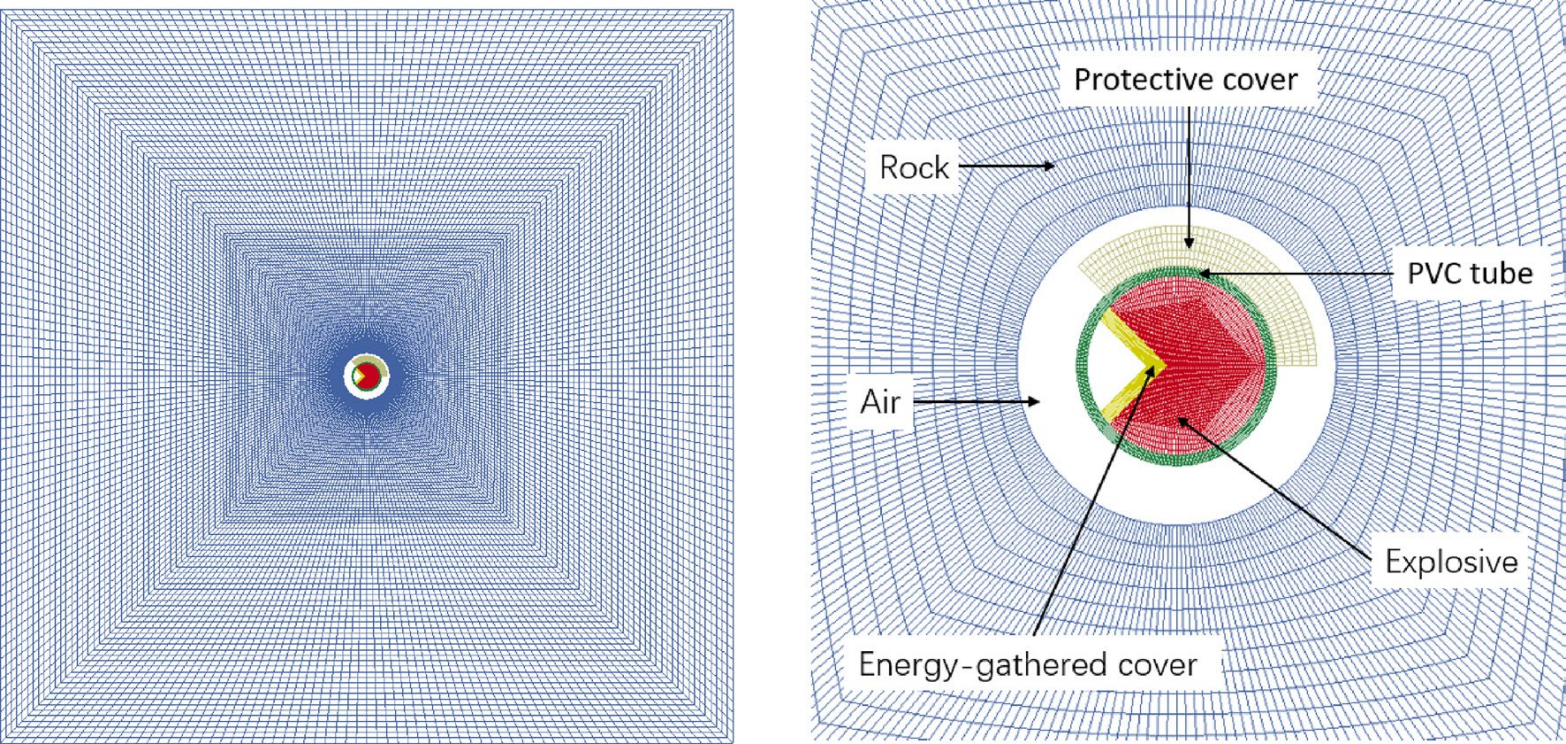
Diagram of model structure.

Drilling and blasting processes of directional damage-reduction shaped charge are complex and rapid. In order to determine the effect of charge structure in blasting process, LS-DYNA software was used for the calculation of the penetration process of PVC charge with thickness 4 mm. Failure keyword MAT_ADD_EROSION was introduced to simulate concrete blasting damage. When concrete unit was subjected to a tensile stress of 1.54 MPa or compressive stress of 17.4 MPa ^40^, unit failure was deleted. The following six materials were considered in the calculation model: explosives, PVC tubes, PVC dissipation materials, red copper energy-gathered covers, cement target plates and air. Among them, explosives, air and copper used Euler grid and ALE algorithm was able to effectively track material structure, which was more suitable for blasting numerical simulations ^41^. Therefore, multi-material ALE algorithm was applied to the three material units. Lagrangian grid was utilized for PVC tubes, P VC dissipation materials, and cement target boards. Fluid–solid coupling algorithm was adopted for the contacts among PVC tubes, PVC dissipation materials, cement target plates, explosives, air, and copper and the entire calculation was performed using cm-g-μs unit system. In order to decrease calculation load, a two-dimensional single-layered solid grid model was developed, which reduced model size, saved calculation time, and made full use of multi-substance ALE algorithm in LS-DYNA. Figure 11 illustrates the model structure.

In simulations, the main charge was considered by *MAT_HIGH_EXPLOSIVE_BURN and Jones-Wilkins-Lee state equation was applied for the determination of functional relationships among the pressure, volume and energy of explosive products during blasting process. Table 5 lists relevant material parameters of explosive ^42^. Johnson–Cook constitutive model and its Gruneison state equation were employed for copper cover and Table 6 presents relevant material parameters ^43^. MAT_PLASTIC_KINE_MATIC constitutive model was applied to both PVC energy-gathered tube and PVC dissipation material and Table 7 shows relevant material parameters ^44^. Air was described by *MAT_NULL material model and its LINEAR_POLYNOMIAL state equation and relevant material parameters ^45^ are given in Table 8. JOHNSON_HOLMQUIST_CONCRETE constitutive model well described the mechanical behaviors of materials under strong dynamic loads. The number of model parameters was small and physical was clear. Therefore, JOHNSON_HOLMQUIST_CONCRETE constitutive model was adopted to describe concrete target plate. Table 9 presents the relevant material parameters of the concrete target plate ^45^.

### Calculation results

**Figure 12:**
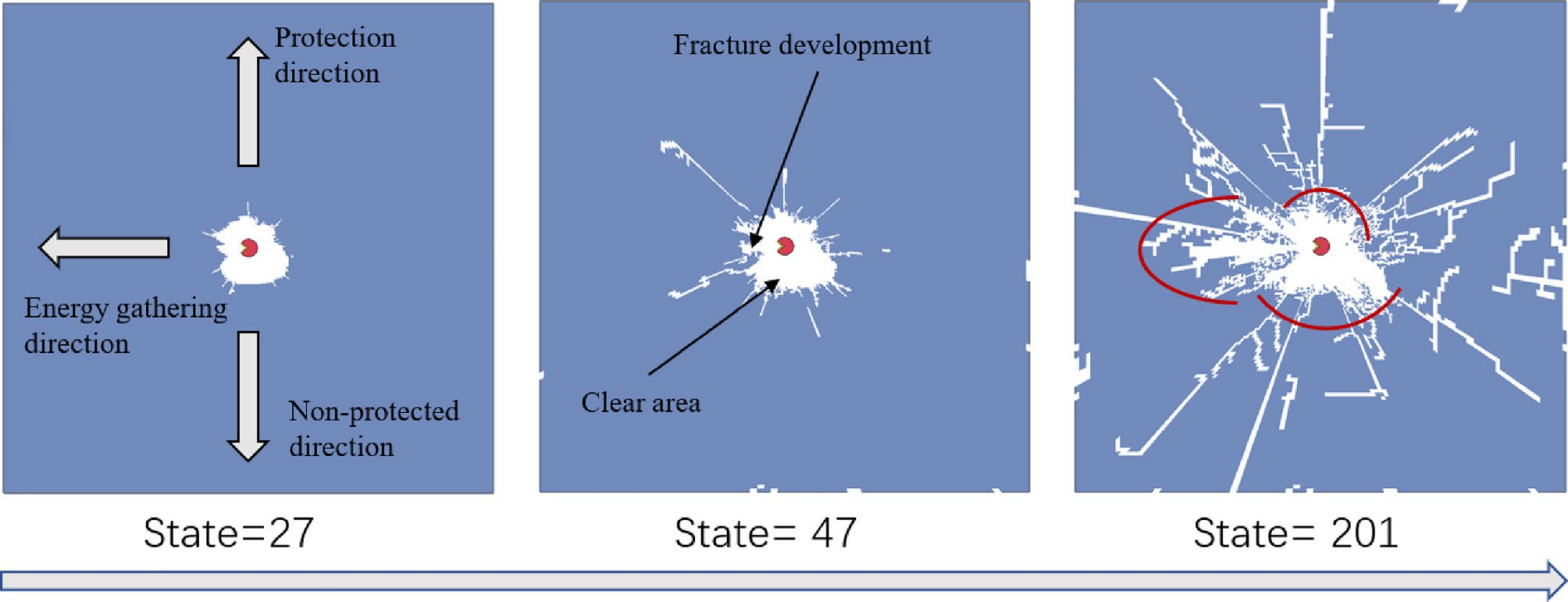
Fracture diagram after blasting.

Figure 12 illustrates model crack development. A clear area was formed around borehole after blasting, which was mainly because the concrete around borehole was broken by high blasting pressure; that is, crushing area. In energy-gathered direction, a main crack gradually expanded from clear area (broken area) along borehole radial direction. The main crack continued to develop along initial expansion direction. The main crack developed regularly and its width and length were larger than cracks along other directions. In non-protected direction, however, clear area was significantly larger than clear area along protection and energy-gathered directions and crack development was more irregular. In protection direction, clear area was smaller, cracks developed finely, and crack lengths were shorter than those along non-protected direction. By analyzing main cracks along energy-gathered direction and comparing crack development degree along protection and the non-protected directions, it was concluded that charge structure was able to directionally control fracture along energy-gathered direction and presented a protective effect on borehole wall along protection direction, which effectively inhibited fracture development.

**Figure 13:**
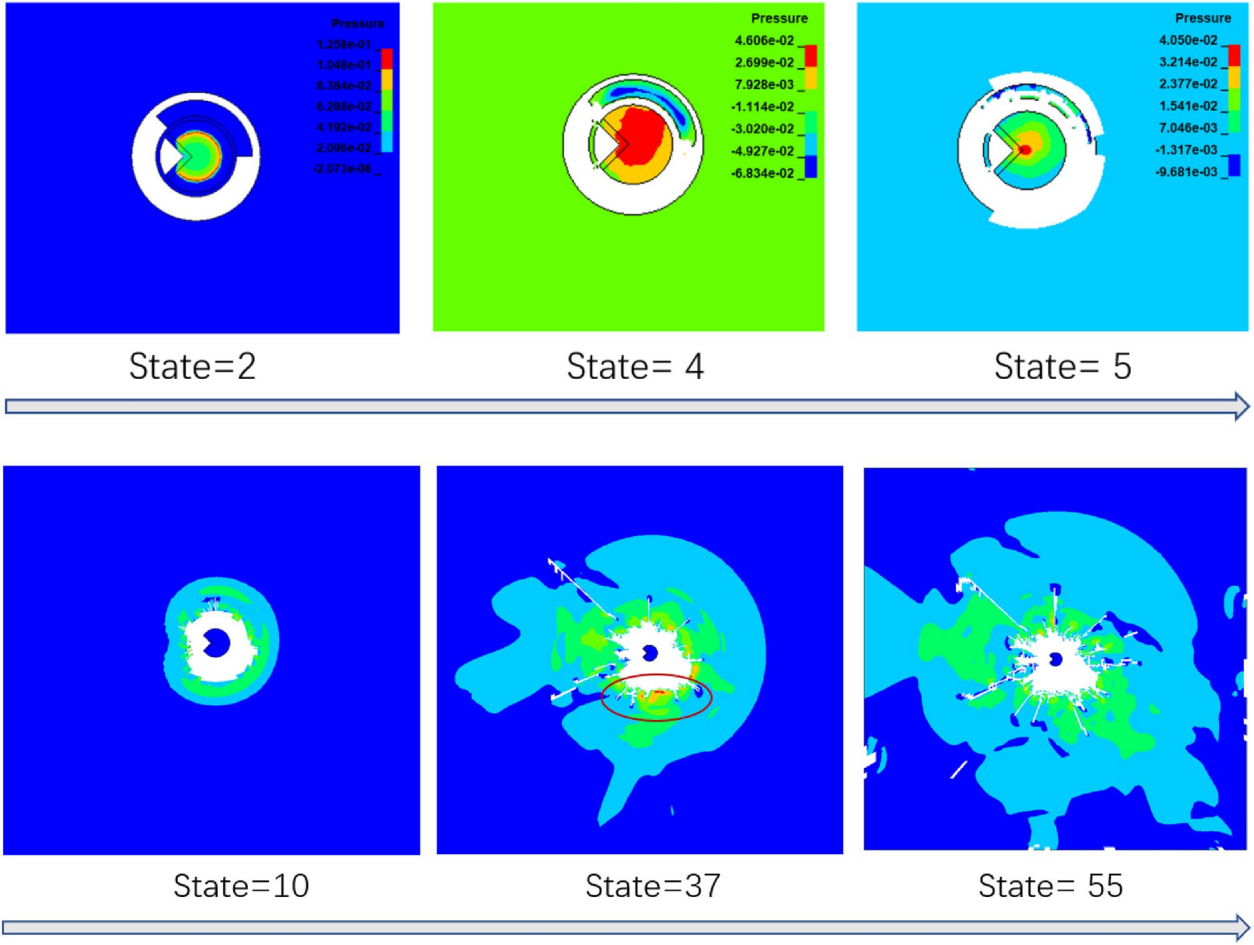
Pressure contour of model.

Figure 13 shows pressure cloud image of target plate penetration process. After blasting, pressure wave along wall-protecting direction first reached PVC dissipation material. It was seen that at State = 4, dissipation material was deformed under force. At the same time, due to uncoupled charge structure along non-protected direction, pressure wave did not reach target plate. Along protection direction, dissipation material absorbed blasting energy and was destroyed. Along non-protected direction, target plate was damaged and damage intensity was greater than that along protection direction. It was concluded that directional damage-reduction shaped charge structure presented obvious damage-reduction influence. At State = 10–55, pressure peak along protection direction was obviously smaller, pressure wave transmission speed was lower, and clear area was smaller than those along energy-gathered and non-protected directions. The peak value along energy-gathered direction was smaller than that along non-wall-protecting direction, but pressure wave propagated the fastest and relatively regular and continued to propagate along energy-gathered direction. Clear area was the farthest from target plate center and was more concentrated. The pressure peak was the largest along non-protected direction, but pressure waves were scattered and irregular. Clear area was large and far away from target plate center.

## Directional rock breaking and reduction mechanism of directional damage-reduction shaped charge

Directional damage-reduction shaped charge structure was consisted of two parts: energy-gathered structure and dissipation material. Dissipation material cover was close to the outside of energy-gathered tube. After the explosion of main charge, when explosive wave propagated inside and reached tube wall, metal energy-gathered cover was crushed, energy-gathered cover elements moved along the axis, and metal particles moved along the axis to form a metal jet ^46^, causing borehole wall directional blasting.

**Figure 14:**
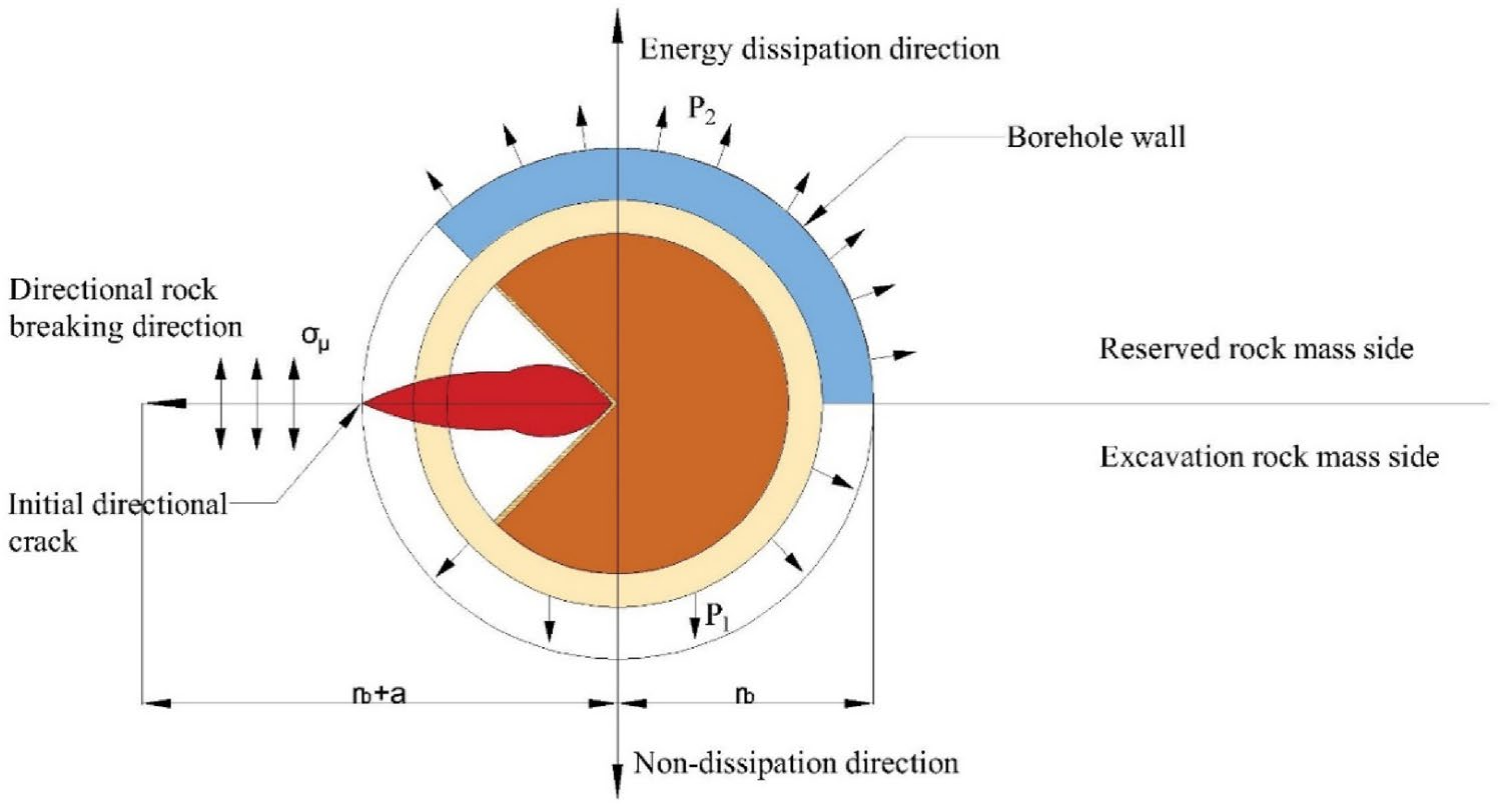
Mechanical model of directional damage-reduction shaped charge.

Metal jet penetration process was divided into three stages: excavation, quasi-steady and termination stages ^47,48^. When metal jet and shock wave reached borehole wall, blasting energy generated an initial directional crack on it ^49,50^. Under the joint effect of stress wave and air wedge, the initial crack gradually extended along radial direction and crack tip accelerated crack extension under pressure concentration and air wedge effects ^51,52^. When shock wave reached the rock on borehole wall, the rock was crushed by compressive pressure which was far greater than its compressive strength, leading to the creation of crushing zone and stress wave propagated far away. At the same time, Poisson's theorem revealed that when the rock was compressed along radial direction, tensile stress occurred along circumferential direction. Concrete is a brittle material whose tensile strength is much less than its compressive strength. When tensile stress was greater than rock tensile strength, circumferential cracks occurred in the rock. Finally, under the combined action of stress and shock waves, crushing zone and circumferential cracks expanded the borehole. According to rock fracture mechanics, a rock breaking dynamic fracture mechanics model with directional damage-reduction shaped charge was developed, as illustrated in Fig. 14.

During rock mass stress-bearing process, rock mass was in a three-dimensional state of mixed tension and compression ^53^. Stress intensity at any point during the period was expressed as:

1 $$\sigma_{i} = \frac{1}{\sqrt 2 }\left[ {\left( {\sigma_{r} - \sigma_{\theta } } \right)^{2} + \sigma_{\theta } - \sigma_{z}^{2} + \sigma_{\theta } - \sigma_{r}^{2} } \right]^{\frac{1}{2}}$$

where $\sigma_{i}$ is stress intensity at any point; ${\sigma }_{r}$ is radial stress intensity; ${\sigma }_{\theta }$ is circumferential stress intensity; and ${\sigma }_{z}$ is vertical stress intensity. When rock mass was exposed to effective stress peak $\left( {\sigma_{i}} \right)_{max} \ge S_{cd}$, a crushing zone was formed. When ${({\sigma }_{{i}})}_{max}\ge {S}_{td}$, a crack zone was also formed, where ${S}_{cd}$ and ${S}_{td}$ are rock mass dynamic compressive and tensile strengths, respectively.

During crack expansion, crack tip stress intensity factor was stated as:

2 $${K}_{1}=PF\sqrt{\pi ({r}_{b}+\alpha )}+{\sigma }_{\mu }\sqrt{\pi \alpha }$$

Since residual tangential stress ${\sigma }_{\mu }$ was much smaller than explosion products pressure P, its influence was ignored ^54^, and Eq. (2) was rearranged as:

3 $${K}_{1}=PF\sqrt{\pi \left({r}_{b}+\alpha \right)}$$

where P is explosion products pressure in the crack, F is stress intensity factor correction coefficient, ${r}_{b}$ is borehole radius, a is crack length, and ${\sigma }_{\mu }$ is tangential stress.

Based on fracture mechanics theory, crack initiation and propagation occurred when K1 > KIC, where KIC is rock fracture toughness. Therefore, to ensure that cracks continued to expand, explosion products pressure had to meet the following conditions:

4 $${\text{P}}>\frac{{{\text{K}}}_{{\text{IC}}}}{{\text{F}}\sqrt{\uppi ({{\text{r}}}_{{\text{b}}+\mathrm{\alpha }})}}$$

Along non-protected direction, explosive wave first compressed the air in borehole, then generating air shock wave which exerted impact load on borehole wall. Borehole wall pressure ${P }_{1}$ under the condition that explosives and rock wall were not coupled was expressed as^55^:

5 $$P_{1} = \frac{{\rho_{e} V_{e}^{2} }}{{2\left( {k + 1} \right)}} \times K_{d}^{ - 2k} \times n$$

6 $${K}_{d}=\frac{{d}_{b}}{{d}_{c}}$$

where ${\rho }_{e}$ is explosive density, ${V}_{e}$ is blasting velocity; k is adiabatic exponent; ${K}_{d}$ is charge non-coupling coefficient; ${d}_{b}$, and ${d}_{c}$ are borehole and charge diameters, respectively; and n is the multiple of pressure increase when detonation air collided with borehole wall.

Along protection direction, explosive wave impact on borehole wall was regarded as a positive impact and approximated as an elastic collision. Borehole wall pressure ${P }_{2}$ under the coupling condition of dissipation material and rock wall was stated as ^56^

7 $${P }_{2}=\frac{{\rho }_{e}{{V}^{2}}_{e}}{k+1}\times T$$

where T is transmission coefficient.

Transmission coefficients T1 and T2 of excavation and protection sides, respectively, were written as:

8 $${T}_{1}=\frac{2{\rho }_{r}{V}_{r}}{{\rho }_{e}{V}_{e}+{\rho }_{r}{V}_{r}}$$

9 $${T}_{2}=\frac{2{\rho }_{f}{V}_{f}}{{\rho }_{e}{V}_{e}+{\rho }_{f}{V}_{f}}\times \frac{2{\rho }_{r}{V}_{r}}{{\rho }_{f}{V}_{f}+{\rho }_{r}{V}_{r}}$$

where ${\rho }_{e}$ is explosive density, ${V}_{e}$ is blasting velocity, k is adiabatic exponent, ${\rho }_{r}$ and ${\rho }_{f}$ are rock and dissipation material densities, kg/m3; and ${V}_{r}$ and ${V}_{f}$ are longitudinal wave velocities of rock and dissipation material, respectively, m/s.

From the above discussion, it was concluded that different rock breaking effects occurred along energy-gathered, protection direction and non-protected directions after blasting. At the same time, stress waves were emitted and transmitted at medium interface. The intensity and boundary of reflected and transmitted waves were related to the wave impedance of the medium on both sides. Along energy-gathered direction, metal energy-gathered jet penetrated vertically into borehole wall and formed an initial directional crack, creating directional cracking in borehole wall. Along non-protected direction, explosive products and waves directly acted on borehole wall, effectively breaking the rock and stress wave was transmitted and reflected once. Along protection direction, dissipation material effectively absorbed the energy generated by the explosive, weakened explosive wave peak pressure, and reduced crushing and breaking effects. At the same time, it prevented explosive products from directly impacting borehole wall along protection direction, thereby controlling blasting energy direction. The stress wave generated by blasting underwent two transmissions and reflections; they were first transmitted to dissipation material on the outside of charge and then transmitted to borehole wall after being buffered, thereby protecting rock wall and preventing over-excavation.

## Field application of directional damage-reduction shaped charge

### Design of directional damage-reduction shaped charge

**Figure 15:**
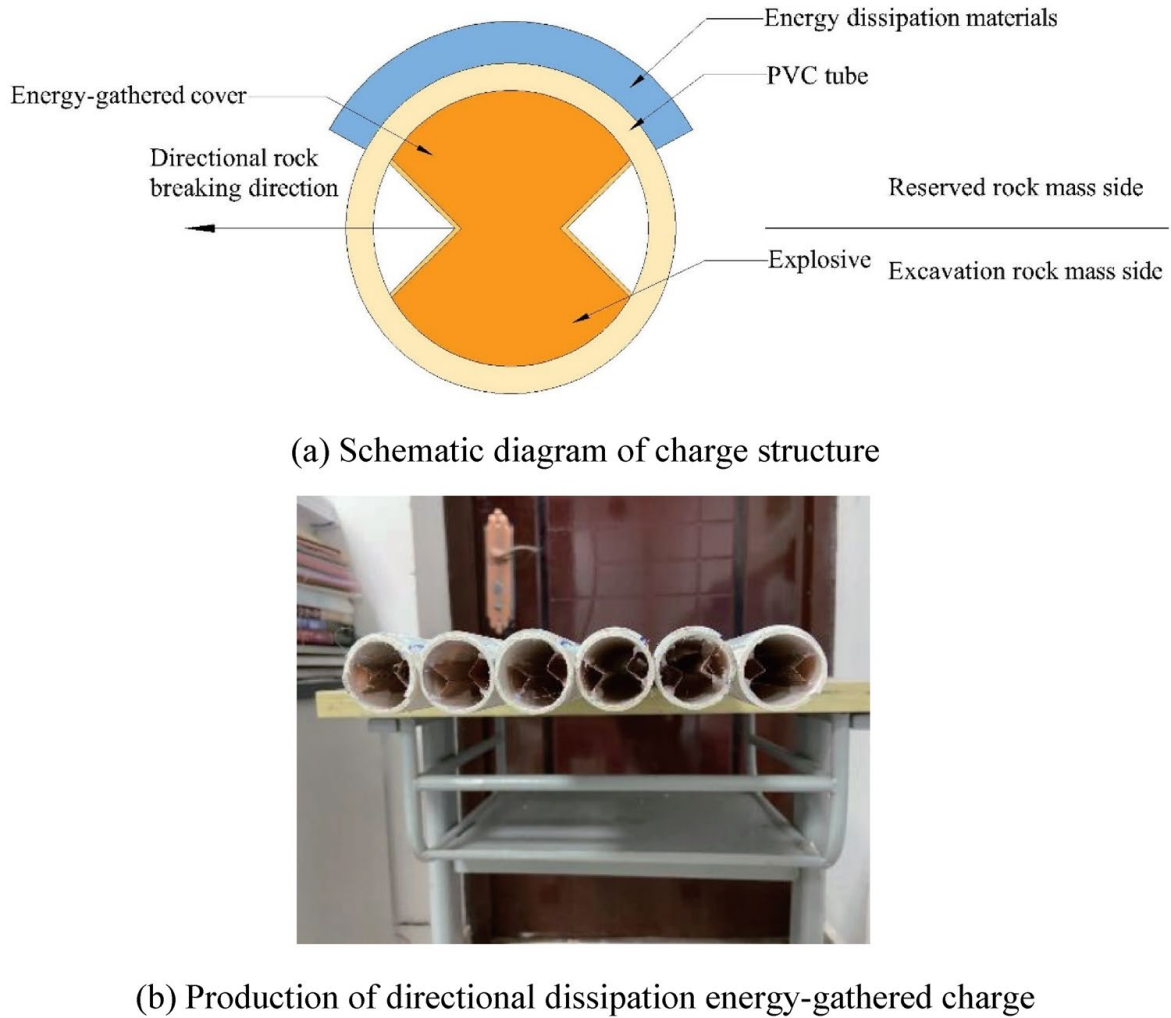
Schematic diagram and production of charge structure.

Deep hole blasting diameter was 40 mm; therefore, designed charge diameter was 32 mm and charge length was 1 m. Red copper was selected as energy-gathered cover 0.5 mm in thickness and cut into copper sheet with a width of 1.4 cm. Then, they were made into triangular energy-gathered covers with angle 90°. Charge tubes were made of PVC, with outer diameter of 32 mm, inner diameter of 28.2 mm, and wall thickness of 1.9 mm. Dissipation material was PVC material which presented the best test effect. Figure 15 illustrates the charge structure.

### Borehole layout and charge

**Figure 16:**
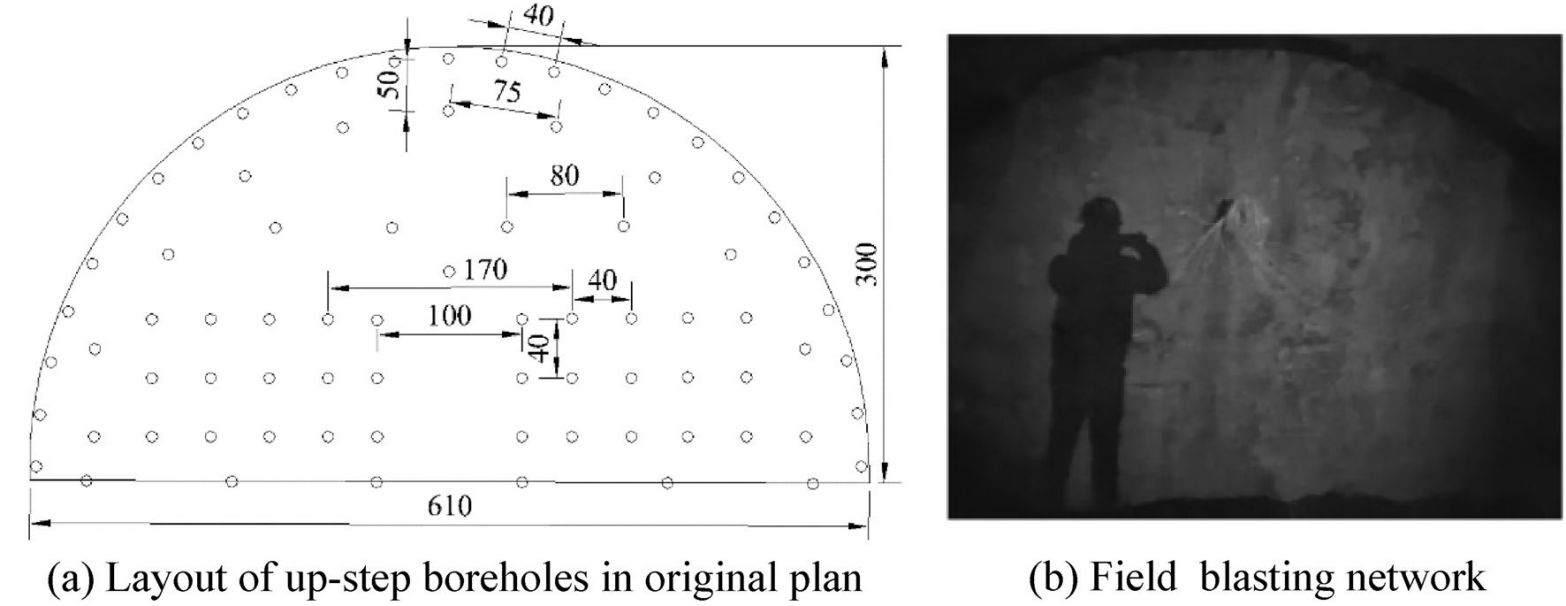
Original blasting plan layout and blasting network.

Field tests were performed in an underground track project in Guiyang City. The engineering geology of this section was poor, rock block saturated uniaxial compressive strength was 30 MPa, surrounding rock lithology was poor, comprehensive classification was level V, and its crushing degree was high. The original blasting plan for the first phase of the project adopted conventional smooth blasting and single-cycle footage was designed to be 1.9 m. Double wedge cutting mode was adopted in this research. A total of 6 pairs of cutting holes were arranged. The distance between the middle two pairs was 1 m and the angle between cutting holes and tunnel face was considered to be about 56°, which was 20 cm deeper than other boreholes, to improve borehole utilization. Figure 16 shows blasting layout and network and Fig. 19 presents blasting effect.

**Figure 17:**
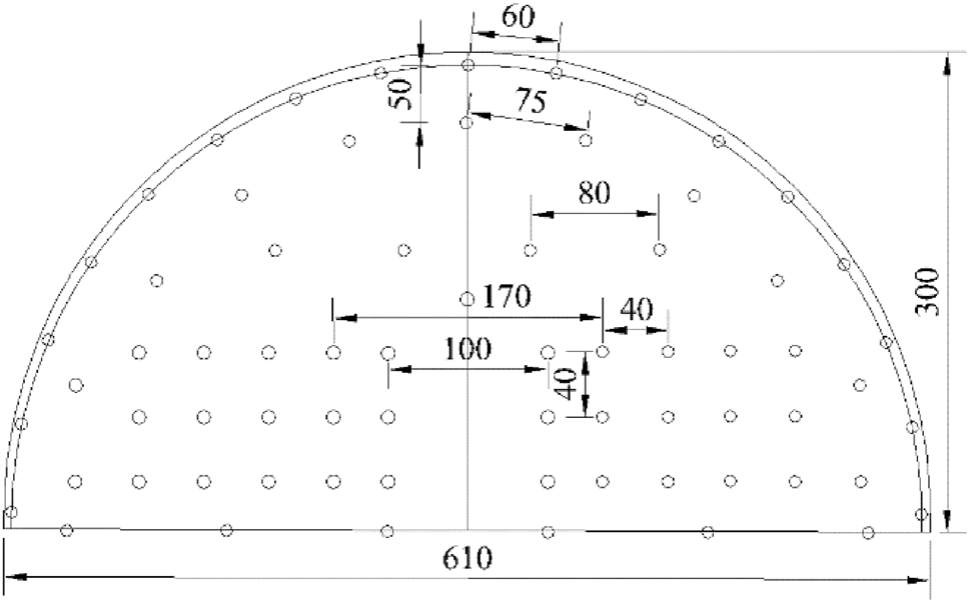
Borehole layout.

**Figure 18:**
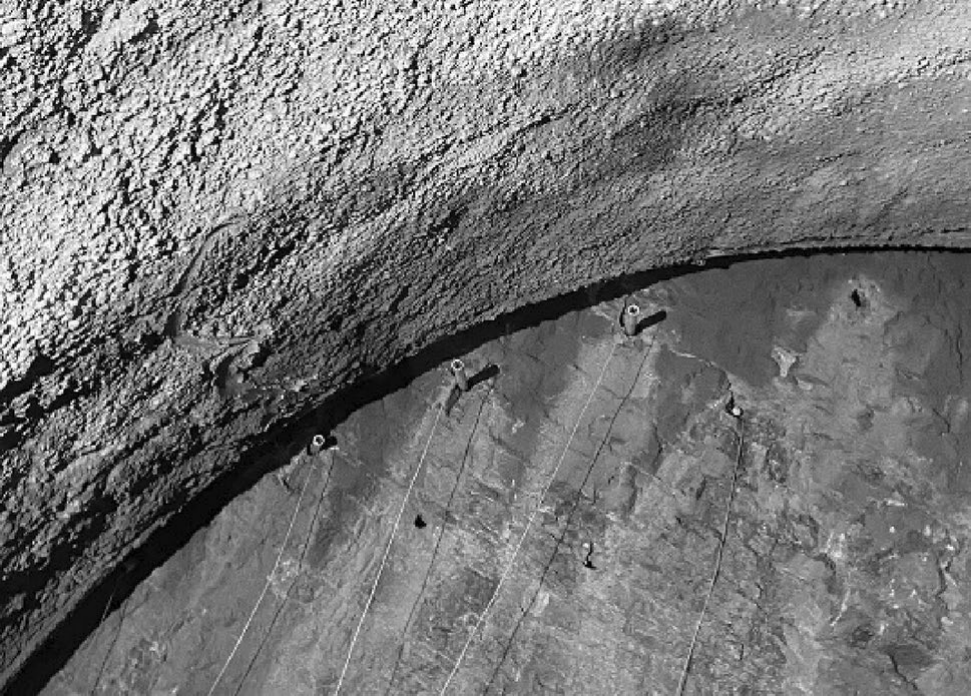
Field charging.

Based on drilling and blasting model test results, it was verified that charge had the ability of directional controlled fracture along energy-gathered direction and formed a better cross-section at the same borehole spacing. Hence, based on the site conditions of the tunnel and combined with existing blasting design, single-hole charge was appropriately decreased during tunnel up step blasting tests. On the basis of site lithology, borehole spacing could be appropriately increased and borehole number could be decreased. Figure 17 illustrates optimized borehole layout and Fig. 18 shows field charge. Also, field test blasting parameters are listed in Table 10. When charging, it was essential to ensure that the energy-gathered direction of each charge as consistent with contour direction and dissipation material was placed toward protection direction.

### Analysis of blasting effects

**Figure 19:**
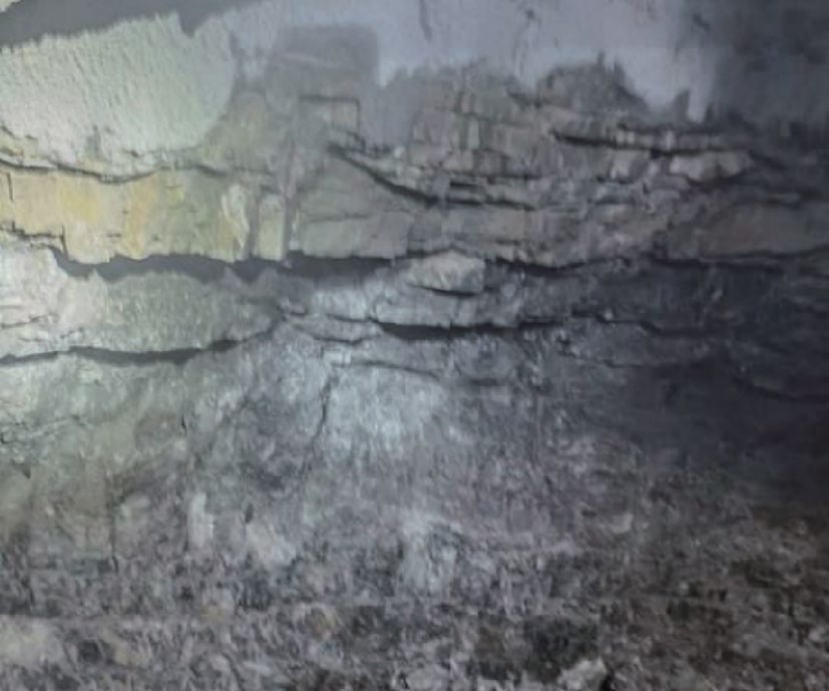
Effect of ordinary blasting.

**Figure 20:**
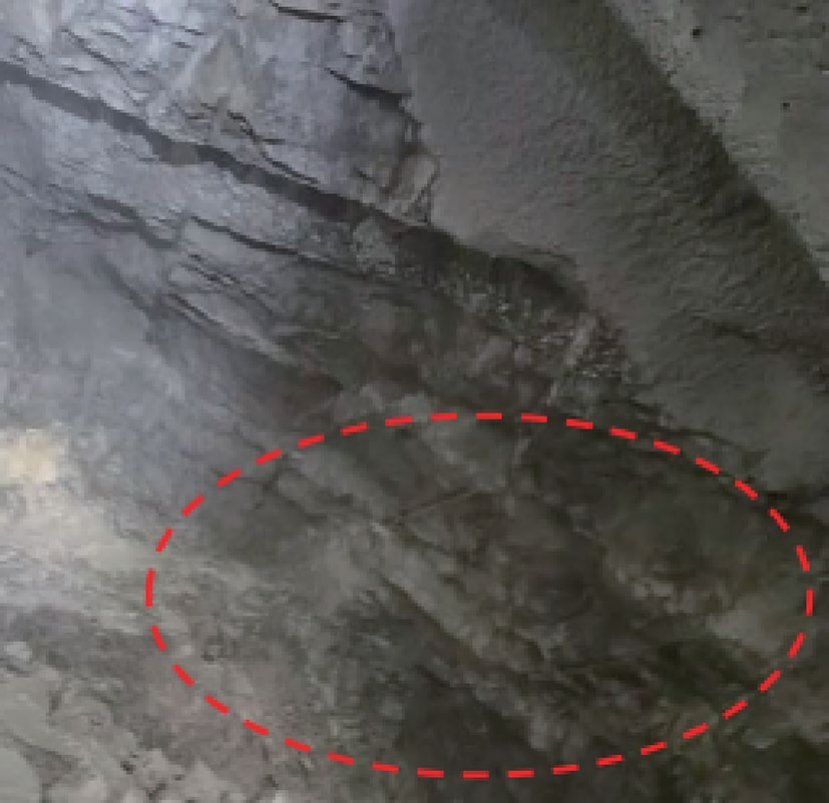
Effect of directional protection energy-gathered blasting.

Figure 20 illustrates cross-section after directional blasting. By comparing Figs. 19 and 20 and combined with statistical results, it was found that there was an obvious cavity around borehole wall after ordinary smooth blasting on up step and crushing area was obvious. Radial random cracks in borehole wall were obvious and there were few half-hole marks. Compared with the ordinary smooth blasting method, when peripheral hole was blasted with directional damage-reduction shaped charge, half hole mark rate was significantly increased, over-under excavation phenomenon was improved to a certain extent, and tunnel contour forming quality and flatness were significantly enhanced. It was concluded that when directional damage-reduction shaped charges were applied to directional rock breaking, tunnel contour quality was improved, fewer cracks formed in borehole wall, and disturbance and damage to rock mass on protection side were also decreased, which improved surrounding rock stability and reduced support time and cost.

Table 11 summarizes post-blasting technical indicators of directional damage-reduction shaped charge blasting and ordinary smooth blasting. Comparison of the obtained results revealed that since the cracks in the excavation section of this project were relatively developed and contained inclined layers, blasting quality could be greatly improved using directional damage-reduction shaped charge blasting. At the same time, there were obvious half-hole marks under these working conditions, with half hole mark rate of about 45%. Over-under excavation amount was decreased by about 2 times. Compared with traditional charge smooth blasting, directional damage-reduction shaped charge presented a significant advantage in directional rock breaking.

**Table 11:** Comparison of post-blasting indicators of two blasting methods.

Technical indicator	Number of peripheral hole (piece)	Drilling time (min)	Secondary cracks in borehole wall	Half hole mark (strip)	Half hole mark rate (%)	Average over-under excavation (cm)
Ordinary blasting	25	150	Obvious crushing around borehole wall	3	12	± 32
Directional dissipation shaped blasting	20	120	Slightly-crushed surrounding rock and few cracks	9	45	± 18

## Conclusion and discussion

This research applied model tests, numerical simulations, theoretical analyses and other techniques to investigate the influences of different dissipation materials on the blasting effects of directional damage-reduction shaped charges and the following conclusions were drawn.

1. A high-speed camera was applied for model tests to observe instantaneous energy trends of dissipation shaped charge. Dissipation material hindered explosion products outward diffusion, indicating that its structure had certain dissipation ability and was protective to borehole wall. Model crack characteristics revealed that charge structure had a directional effect on blasting energy and could break rock in a directional manner.
2. Strain data showed that positive and negative strain peak values at measuring point 4 in specimen 1 were only 0.27 and 0.66 times those at measuring point 9, respectively. The peak values of positive and negative strain at measuring point 4 in specimen 2 were only 0.62 and 0.88 times those at measuring point 9, respectively. The peak values of positive and negative strain at measuring point 4 in specimen 3 were only 0.44 and 1.09 times those at measuring point 9, respectively. In the three experiment groups, the positive and negative strain peaks along protection direction were significantly lower than those along non-protected direction, indicating that the charge structure could effectively absorb blasting energy. It was also concluded that PVC charge presented a better damage-reduction effect.
3. According to the test results of 4 mm-thick directional damage-reduction shaped charges with three different dissipation materials and combined with the analysis of high-speed photography videos, strain data and model crack properties, it was found that PVC charge presented the best directional rock-breaking and rock protection effect.
4. Numerical simulation results showed that clear area along protection direction was significantly smaller than that along non-protected and energy-gathered directions. Cracks along protection direction were finely developed and crack length was shorter than those along non-protected and energy-gathered directions. Dissipation materials effectively absorbed blasting energy, prevented explosive products from directly impacting reserved rock masses, hindered crack expansion in reserved rock masses, and presented obvious damage-reduction effects.
5. Directional rock breaking and dissipation mechanisms of directional damage-reduction shaped charges were discussed according to the obtained experimental and numerical simulation results. Jet could directionally break rock mass and dissipation material attenuated blasting stress waves. When the explosive materials exploded, high temperature and high pressure deformed dissipation material, absorbing part of blasting energy and resulting in stress peak dissipation and prolonging blasting stress wave propagation time.
6. Field test results revealed that the application of directional damage-reduction shaped charge for controlled blasting in tunnels presented obvious excavation contour control effects. Compared with ordinary smooth blasting methods, all technical indicators were improved, half hole mark rate was increased by 33%, and the amount of over-under excavation was decreased by about 2 times.

In summary, the directional loss-reducing aggregation charging structure through the aggregation of energy cover will gather energy directed to break the rock, energy-dissipating materials to protect the surrounding rock, reduce the scattering material produced by blasting, the technology is relative to the traditional blasting technology to improve the efficiency of the traditional method, improve the safety of blasting, while reducing the damage to the surrounding environment. Tests have shown that the directional loss of aggregation charge structure energy dissipation and aggregation effect is obvious, can effectively protect the surrounding rock and directional rock breaking, of which the PVC directional loss of aggregation packets have the best effect, can be used in mining, tunneling and other engineering applications. However, this test only studied the material of energy dissipation cover, there are still deficiencies in the existing research, and further research can be done on the thickness of energy dissipation cover in the future research.

## Data Availability

Data supporting the results of this study are available from the corresponding author [Yiping Zhang] upon reasonable request.
